# Supplementary material for: Earth system instability amplified biogeochemical oscillations following the end-Permian mass extinction
Source: Nat Commun. 2025 Apr 18;16:3703. doi: 10.1038/s41467-025-59038-0 (PMC12008425; doi:10.1038/s41467-025-59038-0)
Supplement: Supplementary file 1 — Supplementary Information [file 41467_2025_59038_MOESM1_ESM.pdf]

Supplementary Information for

Earth system instability amplified biogeochemical oscillations following the end-Permian mass extinction

Ziheng Li[1, 2], **Timothy M. Lenton**[2], **Fei-Fei Zhang**[3], **Zhong-Qiang Chen**[1*], **Stuart Daines**[4]

[1] *State Key Laboratory of Geomicrobiology and Environmental Changes, China University of Geosciences, Wuhan 430074, China*

[2] *Global Systems Institute, University of Exeter, Exeter, EX4 4QU, UK*

[3] *School of Earth Sciences and Engineering, and Frontiers Science Center for Critical Earth Material Cycling, Nanjing University, 163 Xianlin Avenue, Nanjing 210023, China*

[4] *University of Exeter, Exeter, EX4 4QU, UK*

**Corresponding author Emails:*

zhong.qiang.chen@cug.edu.cn

1. Uppermost Permian to Lower Triassic $\delta^{13}C_{carb}$ database

Carbon isotope compositions of marine carbonate ($\delta^{13}C_{carb}$) are most commonly considered as a proxy to reflect the changes in the relative export/burial rates of inorganic vs. organic carbon[1-2]. As a result, secular $\delta^{13}C_{carb}$ profiles have been widely used for chemostratigraphical correlations[3-5]. Here, a total of 7491 $\delta^{13}C_{carb}$ data from 47 Permian-Triassic sections were compiled (Fig. S1; Table S1).

The age model of these sections is based on conodont biostratigraphic and carbon isotope stratigraphic correlations[4], and the age-tied points are listed in Table S2.

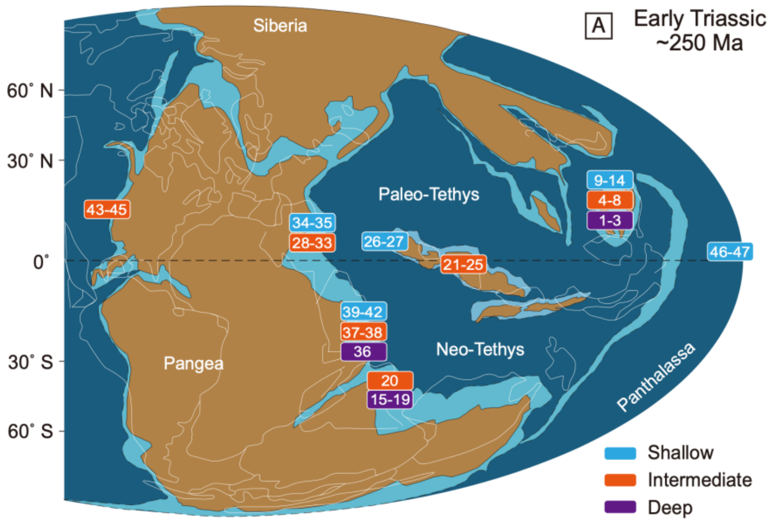

Figure S1. Early Triassic palaeogeography (ca. 250 Ma) and locations of these 47 sections reviewed here. See Table S1 for details of section names and references.

1.1. South China sections (eastern Paleo-Tethys)

The South China Craton Fwas situated at low northern paleolatitudes (~15–30°N) in the eastern Paleo-Tethys Ocean during the Early Triassic (Fig. S1). The central part of the South China Craton was occupied by the Yangtze carbonate platform (Fig. S2), and it was bordered to the north by a carbonate to siliciclastic deepwater ramp that faced into the eastern Paleo-Tethys. The southwestern margin of the craton was occupied by the Nanpanjiang Basin, which comprised a series of shallow carbonate platforms separated by deepwater areas accumulating radiolarites and claystones[6] (Fig. S2).

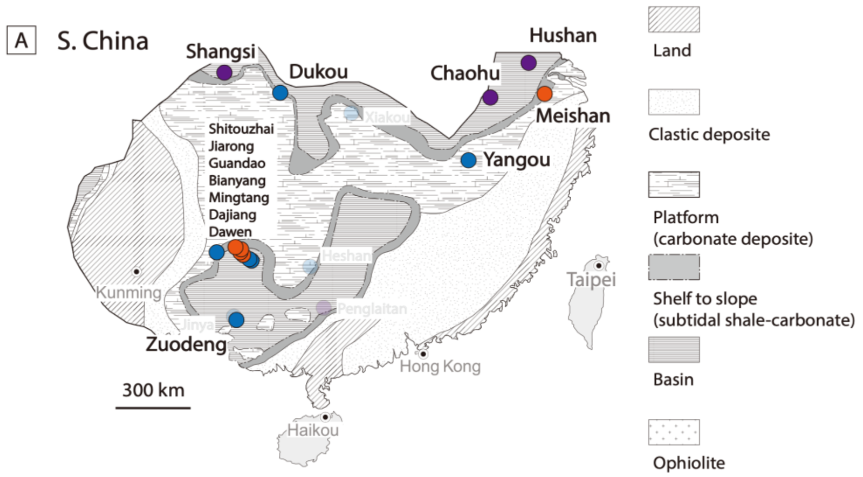

Figure S2. Locations of the uppermost Permian to Lower Triassic sections in South China[6].

Here the sections include: (1) five deep to shallow water sections from northern margin: Shangsi, Hushan, Chaohu, Meishan, and Dukou sections[7-8]; (2) one shallow water section: Yangou section on the Yangtze platform[9]; (3) eight deep to shallow sections from the Nanpanjiang Basin: Guandao, Bianyang, Mingtang, Shitouzhai, Zuodeng, Dawen, Dajiang, and Jiarong sections[6,8-9,10-11] (Fig. S2).

1.2. Sections from southern Neo-Tethys and central Paleo-Tethys

The Permian-Triassic sections from the southern Neo-Tethys sections include: the Tulong section from the southern Tibet of China[12], several sections from the Spiti Valley, and the Losar section from Himachal Pradesh, northern India[12-15], the Guryul Ravine section from Kashmir, northern India[16], and the Surghar and Salt Range sections from the Daud Khel region, Pakistan[17] (Fig. S3). Five sections from the central Paleo-Tethys include the Kuh-e-Ali Bashi, Zal, Abadeh, Shahreza, and Kisejin sections of Iran[18-22] (Fig. S3).

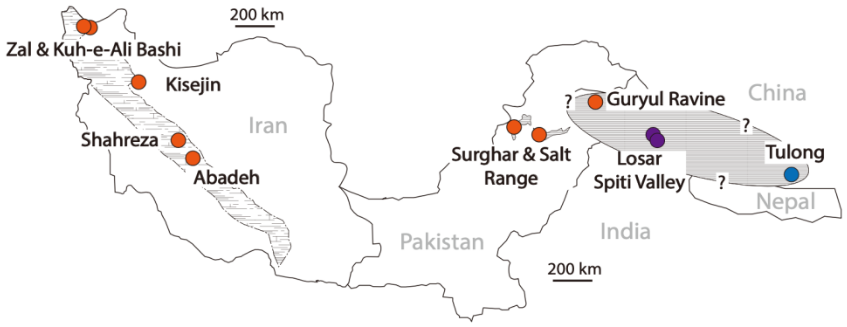

Figure S3. Locations of uppermost Permian to Lower Triassic sections in the southern Neo-Tethys (southern Tibet of China, India, and Pakistan) and central Paleo-Tethys (Iran).

1.3. Sections from western Tethys

As shown in Fig. S4, there are two sections from Turkey: Taskent and Curuk dag section[23]. Other eight sections from Europe include: the Bálvány North and Bálvány East sections from Hungary[24], the Bulla, Uomo, Lungenfrischgraben, and Trudener Bach sections from Italy[25], the GK-1 section from Austria[26], the Plavno section from Croatia[27], and the Idrija-Ziri section from Slovenia[28].

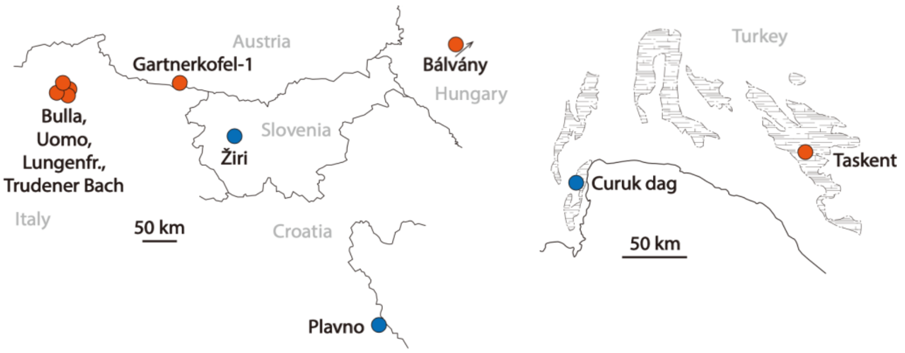

Figure S4. Locations of the uppermost Permian to Lower Triassic sections in western Tethys.

1.4. Sections from southwestern Tethys

Seven sections from Oman and UAE are reviewed here, including the Radio Tower, Wadi Maqam, Wadi Wasit (South), Baid (Wadi Alwa), Wadi Sahtan, and Musandam sections[29-31] (Fig. S5).

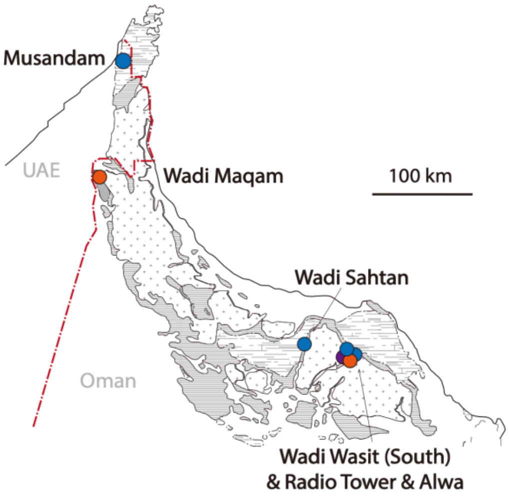

Figure S5. Locations of the uppermost Permian to Lower Triassic sections in the southwestern Tethys.

1.5. Sections from Panthalassa

A total of five sections from the Panthalassa or West Pangea have been reviewed, including the Kamura section from Japan[32], Hot Springs, Spruce Mountain and Confusion Range from USA[33-35] (Fig. S6), and Jesmond section from Canada, which is not shown in figure[36].

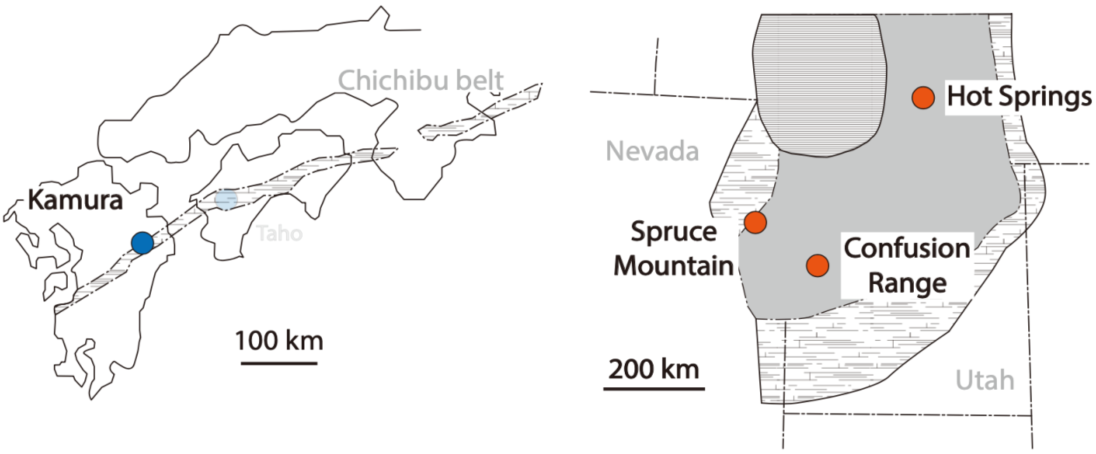

Figure S6. Locations of the uppermost Permian to Lower Triassic sections from Panthalassa.

2. End-Permian to Early-Triassic $\delta^{238}U_{carb}$ and $\delta^{18}O_{apatite}$ database

A total of 468 $\delta^{238}U_{carb}$ data were compiled here, including sections from South China (eastern Paleo-Tethys)[23,36,37-38], western and central Paleo-Tethys[19,23,26], and Panthalassa[32]. A total of 616 $\delta^{18}O_{apatite}$ data were compiled[39-42].

3. Biogeochemical model

The biogeochemical Earth system model represents the long timescale ($\geqslant 10^5$-10^8yr) evolution of the global atmosphere-ocean carbon A, oxygen O, ocean phosphorus P and uranium U as a set of coupled ordinary differential equations[43] (Table S3). There is no explicit representation of sedimentary reservoirs, hence the model is restricted to timescales shorter than the sedimentary timescales ($\simeq 10^8$ yr). Model functional forms and parameterizations for the atmosphere, climate and land surface are summarized in Table S3 and constants are listed in Table S4. Functional forms and parameterizations are from the GEOCARB[44] and COPSE[45-46] models. Carbon isotopes are calculated from isotope mass balance for the A reservoir, using the fractionation factors for marine carbonate and organic carbon burial from COPSE[45]. The grid involved ocean and the parameterization that controls the homogeneity of the ocean floor redox conditions are described in the following section.

3.1. Idealized column ocean model

Many previous long-timescale biogeochemical models[47-49] have employed zero or low-dimensional box models to represent the marine environment, which necessarily then requires a parameterization of global marine burial fluxes that combines spatial averaging and local controls. Yet, when the Earth's global surface is viewed as a nonlinear dynamical system, and the flux is excessively parameterized, the system forfeits its dynamic characteristics. Following[43] we use an idealized column ocean model for ocean-floor oxygen and burial fluxes.

The column ocean model provides an abstract representation of the distribution of burial fluxes across various oceanfloor redox environments. It represents the global ocean as a set of columns, where water column oxygen demand varies between columns controlled by per-column parameters k_U^i (the nutrient availability or (in)efficiency of nutrient uptake[44,46]). Conceptually, we order the columns by increasing k_U^i hence decreasing O_{floor}^i (oxygen level for each column's ocean floor).

$$k_U^i = \left(1 - \frac{i}{ncol}\right) * kU_{min} + \frac{i}{ncol} * kU_{max} \quad (S1)$$

Where ku_{min} and ku_{max} are lower and upper limits that control the per-column k_U^i. In this study the ku_{max} is fixed at 1.0, and ku_{min} is set by ku_{min} = S * 1.0. Where S is the new stability forcing which is varying along the time and control the homogeneity of the ocean floor redox condition. ncol = 100 is the number of the columns. We assume that marine export production in each column is proportional to marine phosphorus (P), and that water column oxygen demand and organic carbon burial (F_{Corg}^i) are proportional to export production and hence P.

$$F_{mocb}(P/P_0) = \sum \left(\frac{P}{P_0}\right) * Corgb_f * k_{mocb}^i \quad (S2)$$

Where k_{mocb}^i is per-column marine organic carbon burial. $Corgb_f$ = 1.0 is the multiplier that controls the overall organic carbon burial flux. Ocean floor oxygen in column *i* is then given by the balance between oxygen supply (proportional to atmospheric oxygen) and demand. The local (or per column) redox condition is given by[46]:

$$\frac{O_{floor}^i}{O_0} = \frac{O}{O_0} - k_U^i \left(\frac{P}{P_0}\right) \quad (S3)$$

$$local_{anoxia}^i = \frac{1}{1 + e^{k_{anox} * O_{floor}^i / O_0}} \quad (S4)$$

Where k_{anox} = 100 controls the sharpness of the transition between oxia and anoxia. Phosphorus burial $F_{pburial}$ is proportional to export production and with potential sensitivity to local ocean floor oxygen level O_{floor}^i defined by an oxygen-sensitive C:P burial ratio CP_{burial}^i.

$$CP_{burial}^i = \frac{CP_{oxic} * CP_{anoxic}}{\left(1 - local_{anoxia}^i\right) * CP_{anoxic} + local_{anoxia}^i * CP_{oxic}} \quad (S5)$$

$$F_{pburial} = \sum F_{mocb}^i / CP_{burial}^i \quad (S6)$$

Where CP_{oxic} = 180 and CP_{anoxic} = 180*4, are the C:P burial ratio under oxic and anoxic conditions[47]. The column ocean module allows for various burial environments for phosphorus and organic carbon. This reveals the long-timescale dynamical characteristics of systems based on the phosphorus-oxygen-carbon cycles. The relevant control on the ocean-integrated burial of organic carbon and phosphorus

can be captured by considering k_U as a function of normalized cumulative organic carbon burial. Additionally, the two-sink U model are controlled by the core ***P-O-A*** dynamic system, whilst no extra feedbacks are considered here for U. Hence, in the context of the biogeochemical modeling U are not separate state variables in our model.

3.2. Marine total P burial can be a non-monotonic function of P level

The marine P cycle is closely linked to the primary production and, hence, the source and sink of the marine and atmosphere oxygen level. The redox-sensitive P burial in marine sediments has been explored in multiple previous studies[50-52] including: (1) enhanced P recycling flux from sediments to water column in oxygen-depleted condition, which has potential to enhance the primary production. Greater primary production increase the export of organic carbon to deeper water (> 100 m) and intensifies the oxygen demand in water column, thus strengthen the ocean anoxia. This is recognized as the short-term (~1ky) positive feedback loop; (2) burial of organic carbon in oxygen-depleted depositional environment is the source of atmospheric oxygen, which eventually leads to the increasing atmospheric oxygen concentrations and gradually counteract the decreases in bottom water oxygen. This is recognized as the long-term (~1 Ma) negative feedback loop.

As a reminder, by using the column ocean model described above, we explicitly consider the local redox dependence of the Corg:Ptotal burial ratio which has two competing effects on globally integrated marine burial fluxes: increasing marine phosphorus tends to drive an increase in productivity hence increase phosphorus burial, but also trends to expend marine anoxia which reduces phosphorus burial.

Here we show how the homogeneity of the ocean floor redox condition controls the globally-integrated phosphorus burial flux. We take (ku_{min}, ku_{max}) = (0.1, 1.0) and (0.7, 1.0) for stable and unstable system, respectively (Fig. S7A). More detailed, for (ku_{min}, ku_{max}) = (0.1, 1.0), 100 ocean columns have their nutrient availability varying between a wide range. And for (ku_{min}, ku_{max}) = (0.7, 1.0), all the ocean columns have

their nutrient availability varying between a narrowed range (homogeneous marine burial environments). The fraction of global marine organic carbon burial in anoxic oceanfloor environments gradually and nonlinearly increase in stable system, and sharply increase when the phosphorus level reaches a threshold value in unstable system (Fig. S7B). In the stable system, the gradually increasing of the faction of anoxic oceanfloor gradually increase the Corg:Ptotal ratio from lower oxic value to higher anoxic value, the effect of increasing productivity on burial flux dominates, hence the globally-integrated phosphorus burial flux increase monotonically with increasing marine phosphorus level (Fig. S7C). In contrary, in unstable system, the sharp increase in fraction of anoxic condition results in a sharp increase in Corg:Ptotal ratio. As shown in Figure 7C, (1) when the phosphorus level is below the threshold value, the system stays in stable oxic condition, and Corg:Ptotal ratio fixed at e.g. 180 in our model. The integrated phosphorus burial flux increases monotonically with increasing phosphorus levels. (2) when the phosphorus level is cross the threshold value, Corg:Ptotal ratio sharply increases, and integrated phosphorus burial flux decreases as phosphorus level keeps going higher. In this regime, the effect of increasing anoxia dominates the system. (3) when the phosphorus level is above the threshold value, the system stays in stable anoxic condition, Corg:Ptotal ratio fixed at e.g. 180*4 in our model. The integrated phosphorus burial flux increases with increasing phosphorus level again.

In sum, being controlled by two competing effects, a non-monotonic behaviour of globally-integrated phosphorus burial as a function of marine phosphorus level is observed in the unstable system.

3.3. Stable and unstable phase planes

As stated above, the parameters ku_{min} and ku_{max} controlled the homogeneity of the ocean floor redox condition. The greater difference exists between ku_{min} and ku_{max}, the greater degree of heterogeneity of the ocean floor redox condition is observed.

Geometrically, both ku_{min} and ku_{max} controlled the shape of the critical manifold (the P nullcline surface, where dP/dt = 0)[43].

We show the 3D and 2D phase planes and time-series for a group of tests (Fig. S8). There are two cases: (ku_{min}, ku_{max}) = (0.1, 1.0), (0.7, 1.0), we fixed the ku_{max} and gradually increase the ku_{min} to narrow the difference among them. The systems are set to start from arbitrary initial steady state (***P*** = 0.5, ***O*** = 2.0, ***A*** = 2.5). For the first system (Fig. S8, upper row), when the difference between ku_{min} and ku_{max} is large, systems find their new steady states after model adjustment process. With an increase in ku_{min}, the stability of the system decreases along with the change of the shape of the ***P*** surface. The ***P*** surface (the coloured surface in Fig. S8) gradually folds in ***P-O-A*** 3D-space. For the later unstable system (Fig. S8, lower row), nonlinear features e.g. limit cycle oscillation begin to dominate the behaviour of the system[46].

4. Sensitivity tests of the forcings

The sensitivity tests focus on the forcings VEG, S and Uplift are shown in Figure S10, Figure S11, and Figure S12, respectively. Extended model runs include short-timescale pulses (oxidative weathering, CO2 and phosphorus pulses) (Figs. S13, S14, S15).

Sensitivity tests of the forcing VEG (Fig. S10) include four different vegetation recovery patterns: Partial Anisian recovery, Anisian recovery, gradual recovery, Carnian recovery[53]. Sensitivity tests of the forcing S (Fig. S11) share the same patterns of forcing VEG and Uplift in the main text (Fig. 3). The oscillatory behavior is sustained throughout the early Triassic while the forcing S is maintained at 0.4. Sensitivity tests of the forcing Uplift (U) (Fig. S12) include two groups: (1) constant forcing U (= 0.6, 0.8, 1.0) during the early-Triassic. (2) Uplift values decay from early most Triassic (U = 0.8) to 0.6 and 0.4 at 237Ma, respectively.

5. Extended model runs with short-timescale forcings

The first group of extended model runs (Fig. S13) with short-timescale pulse of oxidative weathering and CO_2 pulse to the atmosphere is applied by using the following functions:

F_{oxidw} = [-1e30, -252e6, -251.999e6, -251.998e6, -251.997e6, -237e6],

[1, 1, O_{ramp}, O_{ramp}, 1, 1]

F_{CO2} = [-1e30, -252e6-1, -252e6, -251.5e6, -251.5e6+1, -237e6],

[1, 1, CO2pulse, CO2pulse, 1, 1]

Here the first vector is the time in years, and the second shows flux multiplier or flux at these times. O_{ramp} (= 100) is the relative rate of oxidative weathering to model the transient enhancement of the oxidation of terrestrial biomass suggested by[54]. CO2pulse is the additional CO_2 flux (= 0.5, 2.0, 5.0e12 mol/yr) injected by Siberia Traps[55]. As revealed by previous studies, the short-timescale pulses can capture the very first $\delta^{13}C_{carb}$ excursion, but can not capture the early Triassic oscillations.

The second group of extended model runs (Fig. S14) involves short-timescale pulse of oxidative weathering (share the same function as above). And the phosphorus pulse to the ocean:

F_p = [-1e30, -252e6-1, -252e6, -251.7e6, -251.7e6+1, -237e6],

[1, 1, Ppulse, Ppulse, 1, 1]

Where the Ppulse is the additional phosphorus flux to the ocean (= 1.0, 2.1, 2.5 mol/yr). The eruption of the Siberia Traps might have triggered wide-spread wildfire[56-58]. Based on the present studies, wildfire (e.g. Australia fire) plays role of enhancing nutrient input to the ocean, positive relationship between wildfire magnitude and marine phytoplankton production is established in a fire-prone region of Kimberley coast[59-60]. Therefore, revealing a stand along biogeochemical process that wildfire increase the new production and drives ocean anoxic event at short-timescale.

The addition of the short-timescale phosphorus pulse predicts an ocean anoxic event around 252 Ma. Combined with enhanced oxidative weathering, the model results match up well with C-O-U isotope values in the unstable system.

6. Extended model run with nitrogen cycle

The third group model run (Fig. S15) activates the nitrogen cycle with fixed forcings VEG (=0.0), S (=0.4) and Uplift (=0.8) to evaluate the P-O-A-N source-sink dynamics in an unstable system. The functional forms of the nitrogen cycle are following COPSE model[46]. We assume that nitrogen fixation compensates for denitrification which is evidenced by the micobialites records that during the early most Triassic pyrite framboids (presenting oxygen-depleted watermass) were only confined to micobialites and absent in other habitats (nonmicrobialite sections)[61]. Indicating that the N-fixing phytoplankton is abundant in anoxic compare to oxic phases and nitrogen fixation rate is greatly enhanced during ocean anoxia to balance the enhanced denitrification.

When nitrogen cycle is considered in the model, the new production (newp) is controlled by both nutrients phosphate and nitrate potentially limiting:

$$\text{newp} = \text{r}_{C:P} * \min\left(30.9 * \frac{\boldsymbol{n}}{\text{r}_{N:P}}, 2.2 * \boldsymbol{p}\right) \qquad (S7)$$

the values 30.9 and 2.2 are the present average concentrations of nitrate and phosphate in the ocean (in μmol/kg). $r_{C:P}$= 117 and $r_{N:P}$ = 16 are the Redfield ratios, and therefore N is proximately limiting at present giving $newp_0$ = 225.96 as a normalizing constant. The **n** and **p** are the nitrate and phosphate reservoir size normalized to the present level.

$$\text{denit} = \text{k}_{denit} * \left(1 + \frac{anox}{1 - \text{k}_{\text{oxic}}}\right) * \boldsymbol{n} \qquad (S8)$$

where the denitrification (denit) increases as anoxia increases. The parameters k_{denit} (4.3e12 mol/yr) and k_{oxic} (=0.86) are the denitrification constant and the present oxic fraction, respectively. The **anox** is the anoxic fraction of the ocean.

$$\text{nfix} = \text{k}_{nfix} * \left(\frac{\boldsymbol{P} - \frac{\boldsymbol{N}}{\text{r}_{N:P}}}{\boldsymbol{P_0} - \frac{\boldsymbol{N_0}}{\text{r}_{N:P}}} \right)^2 for \frac{\boldsymbol{N}}{\text{r}_{N:P}} < \boldsymbol{P}, else\ 0 \qquad (S9)$$

where the nitrogen fixation (nfix) keeps the nitrate just below its Redfield ratio with phosphate. Therefore, the nfix will and is never be enough to overcome the N limitation. The uppercase bold **N** and **P** are the reservoirs sizes in mol, N_0 (4.35e16) and P_0 (3.1e15) are the present-day reservoirs sizes. Marine organic nitrogen burial is a small term dependent on marine organic carbon burial and fix C:N burial ratio = 37.5[46]: monb = mocb/CNsea. Figure. S15 shows that adding the N cycle does not fundamentally alter the P-O-A dominated dynamic system. Importantly, during the limit cycle oscillation, the P cycle is characterized by source-sink imbalance, leading to sharp changes in oceanic P levels and controls the dynamics of the system, while the N cycle is remaining source-sink balance (or steady state) throughout the whole oscillation. These results agree well with the typical geochemical-view that although the N has always been the limiting nutrient of the primary production (N:P < 16) in our model run, the N cycle reaches equilibrium in 0.5e4 (yr) time scale with reasonably high nitrogen fixation flux, meaning the N cycle can potentially have great impact on the short-timescale perturbations e.g., the geochemical anomalies during the onset of the very first carbon isotope excursion (~251.9 to 251.5 Ma). But on the geological time scale (~1 to 20 Myr), the N cycle can be seen as being in equilibrium throughout the whole Early Triassic, and P becomes the ultimate limiting nutrient and dominates the periodic oscillations of the system.

7. Cell-size dominated nutrient uptake efficiency

Previous studies introduced the relationship between the cell-size and the nutrient uptake efficiency (k_U) as[62-63]:

$$k_u = \frac{\mu_1 * \left(\frac{d}{d_0}\right)^{2-\zeta} * \min(NO_3, PO_4)}{K_1 * \left(\frac{d}{d_0}\right) + \min(NO_3, PO_4)} \tag{S10}$$

where μ_1 (~$2d^{-1}$) is the maximum possible growth rate for the smallest cells of diameter d_1 (2e-7 m). K_1 is the half-saturation constant (e.g., 0.005 mmol N m^{-3}). $\min(NO_3, PO_4)$ is the ambient nutrient concentration (e.g., 0.1mmol N m^3). The exponent ζ (lie between ~2.1 to 3.0, we take the minimum value 2.1 here) yields the decreasing of the cell-size leads to the increasing of the nutrient uptake efficiency.

In our model, the forcing S controls the minimum nutrient uptake efficiency along the water columns, hence we substituting the maximum cell-size of the eukaryotic marine phytoplankton (0.2-30 μm) and cyanobacteria (3-10 μm) to obtain the minimum k_U. For the cell size $d = 30\mu m$ and $10\ \mu m$ gives k_U = 0.14 and 0.38 respectively. Matching up well with the values of the forcing S we set here pre (0.1±0.1) and post (0.4±0.1) the PTB, this also agrees reasonably with our estimate that the tipping point of S between the stable and unstable system is around 0.3 to 0.4 (Fig. 4).

8. Supplementary figures and tables

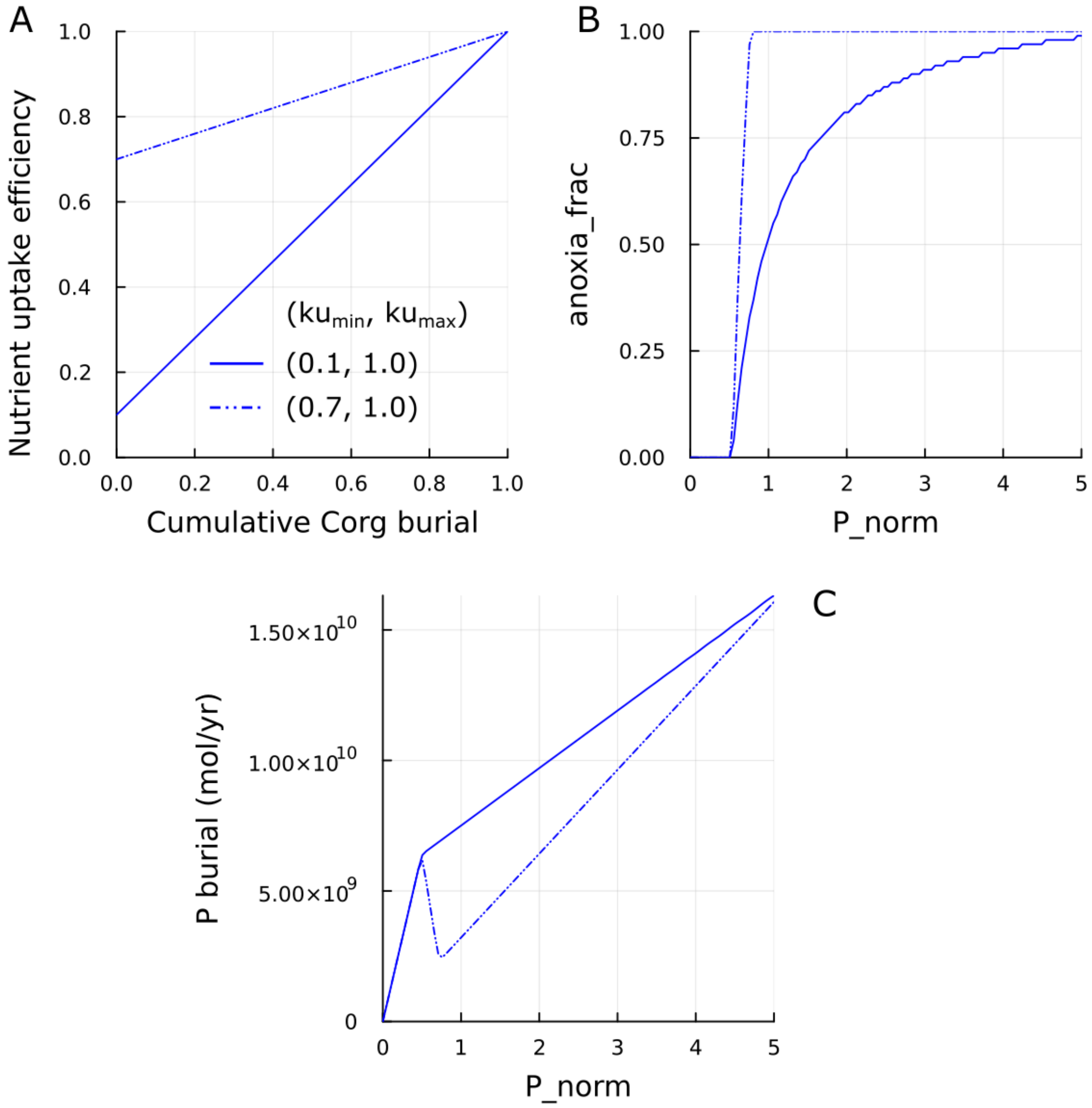

Figure S7. Local redox dependence combines with globally averaged oceanfloor oxygen to determine global marine phosphorus burial. (A) Per-column oceanfloor oxygen demand k_U^i, ncol = 100, (ku_{min}, ku_{max}) = (0.1, 1.0) and (0.7, 1.0) for stable and unstable system, respectively. (B) Fraction of global marine organic carbon burial under anoxic conditions as a function of normalized marine phosphorus level at constant atmosphere-ocean oxygen and carbon. (C) Global marine phosphorus burial as a function of normalized marine phosphorus at constant atmosphere-ocean oxygen and carbon.

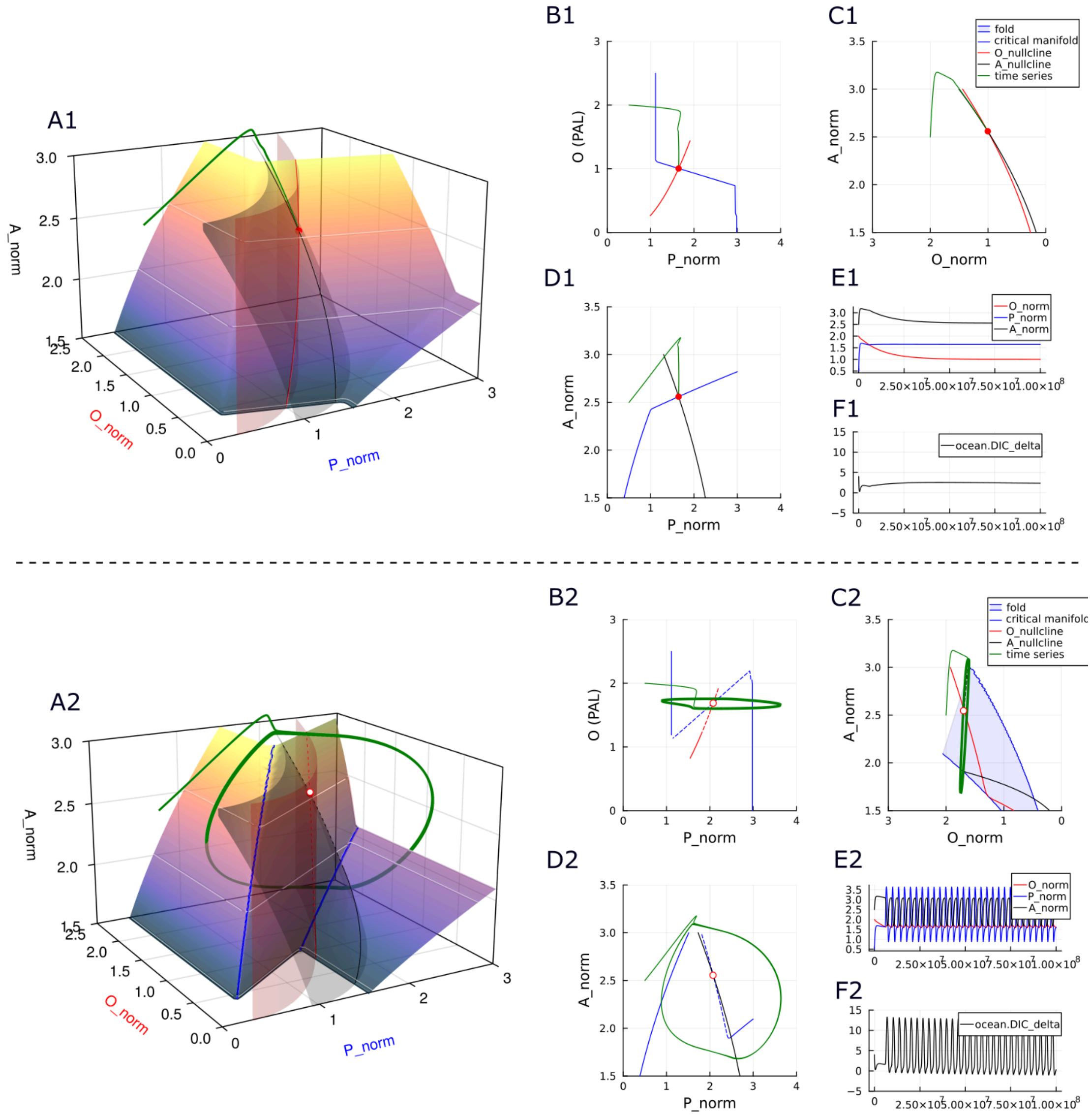

Figure S8. Sensitivity test of (ku_{min}, ku_{max}) = (0.1, 1.0), and (0.7, 1.0), representing stable and unstable system, reespectively. (A1-2) The 3D phase planes, colored surface: P_nullcline surface (critical manifold), red surface: O_nullcline surface, black surface: A_nullcline surface. (B-D) Cross sections in three directions around the equilibrium point (red point). The 3-dimensional ***P-O-A*** surfaces is reduced to lines. (E-F) Time-series of ***P-O-A*** reservoirs and $\delta^{13}C_{carb}$.

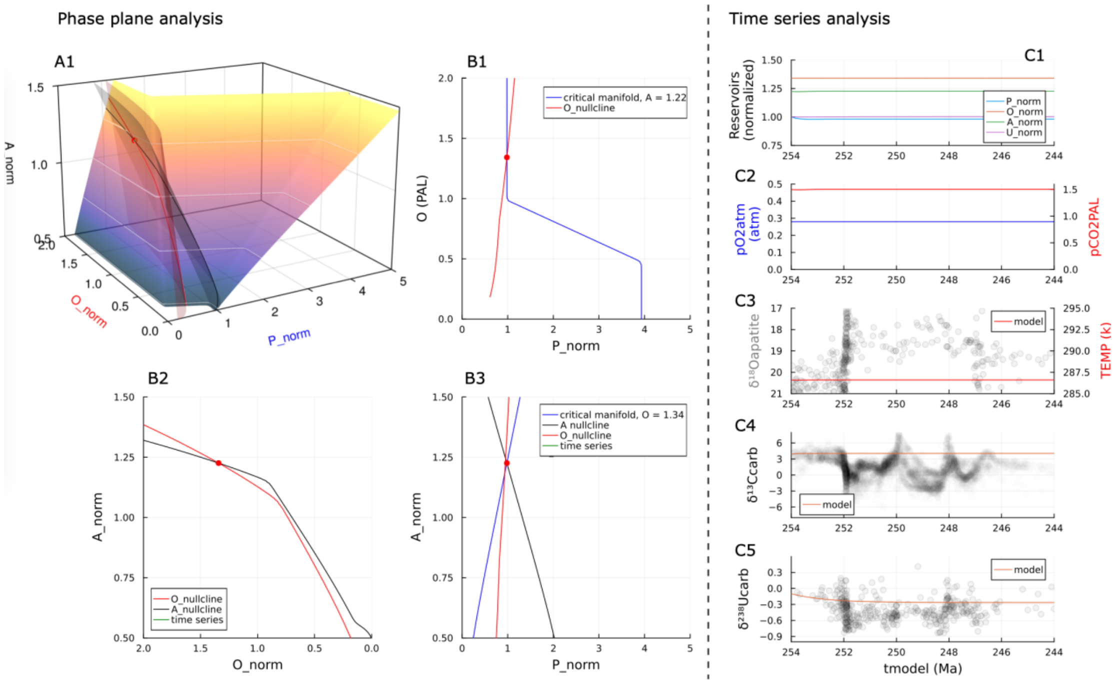

Figure S9. End Permian steady state. Left 3D phase plane (A1) and its mapping to 2D (B1-3). Right, time series analysis: (C1) Normalized values of the reservoirs: phosphorus, oxygen, carbon, and uranium. (C2) pO2 (atm), pCO2 (PAL). (C3) $\delta^{18}O_{apatite}$ (per mil) profile and model predicted Temperature (K). (C4) Dataset and model predicted $\delta^{13}C_{carb}$. (C5) Dataset and model predicted $\delta\,^{238}U_{carb}$.

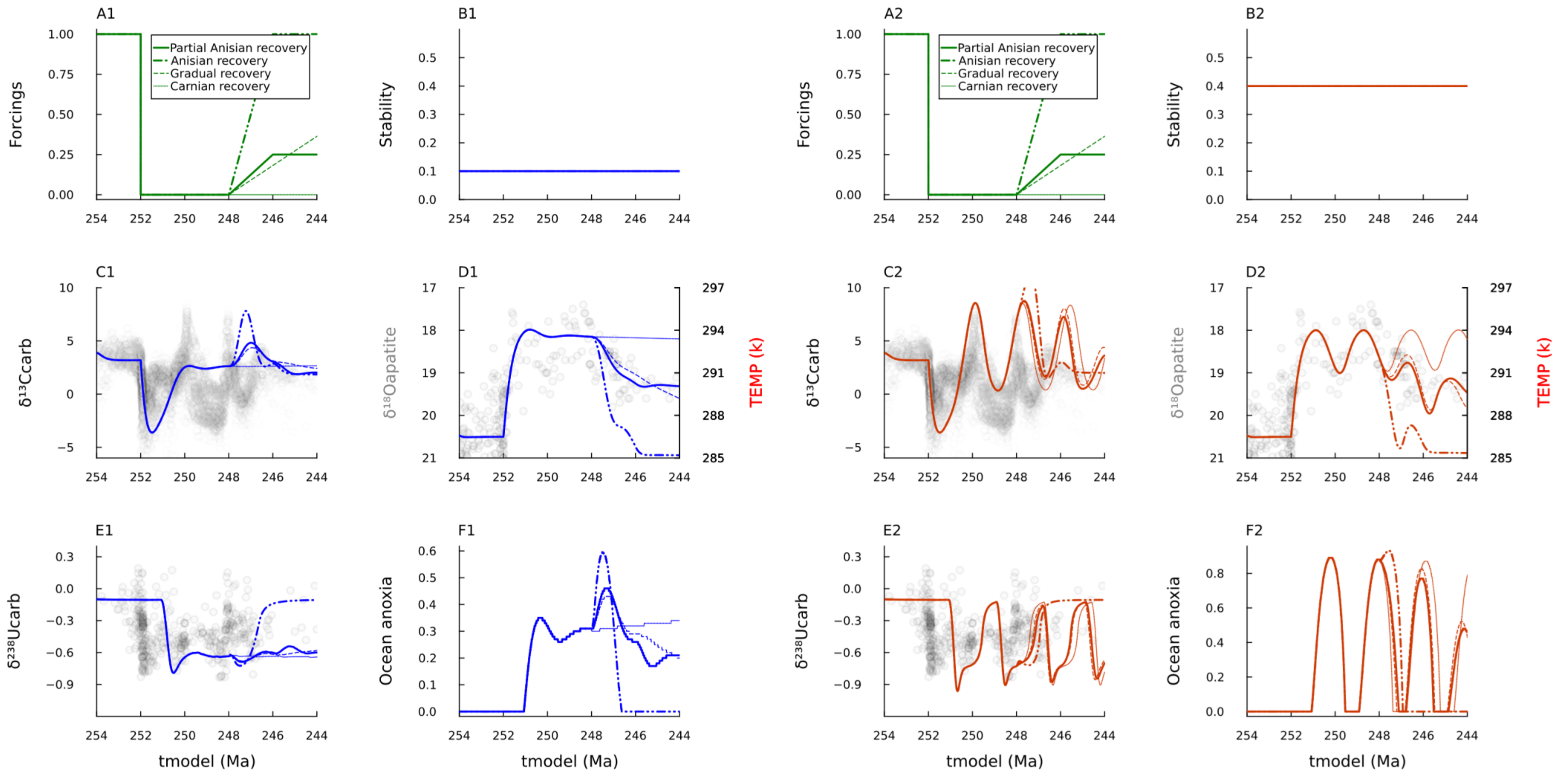

Figure S10. Sensitivity tests of the land plant recovery patterns in stable (left, blue) and unstable (right, red) system. (A1-2) Vegetation (VEG) forcings for 4 different recovery patterns[53]. (B1-2) Stability forcing (S) (C-E) Model-data comparisons of $\delta^{13}C_{carb}$, $\delta^{18}O_{apatite}$ vs. Temperature (K), and $\delta^{238}U_{carb}$. (F1-2) Degree of ocean anoxia. Note the Uplift forcing share the same pattern as in the main text.

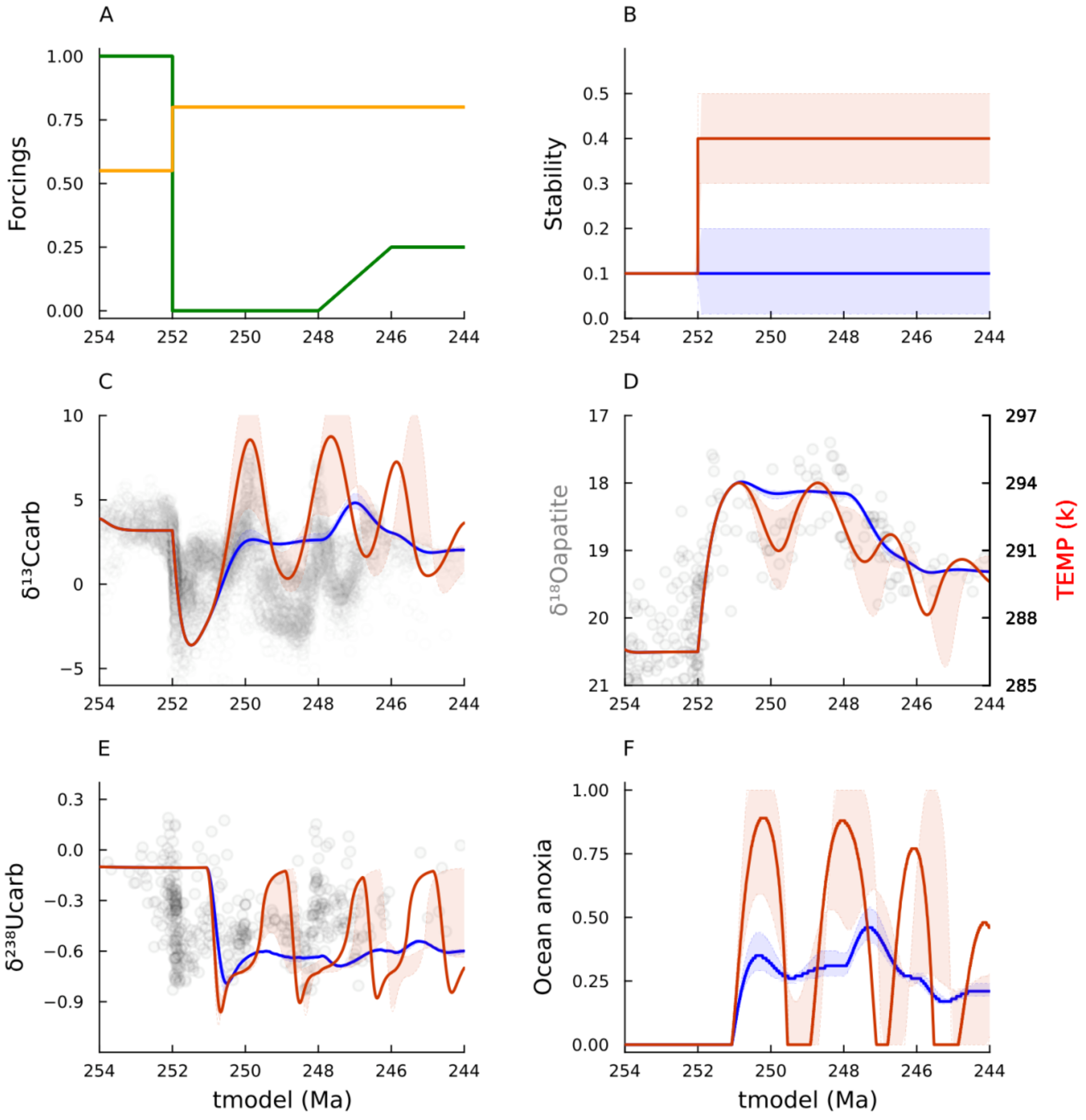

Figure S11. Sensitivity tests of the forcing S. (A) Vegetation (V, green line) and Uplift (U, orange line) forcings. (B) Stability forcing (S). Blue for stable system, red for unstable system. (C-E) Model-data comparisons of $\delta^{13}C_{carb}$, $\delta^{18}O_{apatite}$ vs. Temperature (K), and $\delta^{238}U_{carb}$. (F) Degree of ocean anoxia.

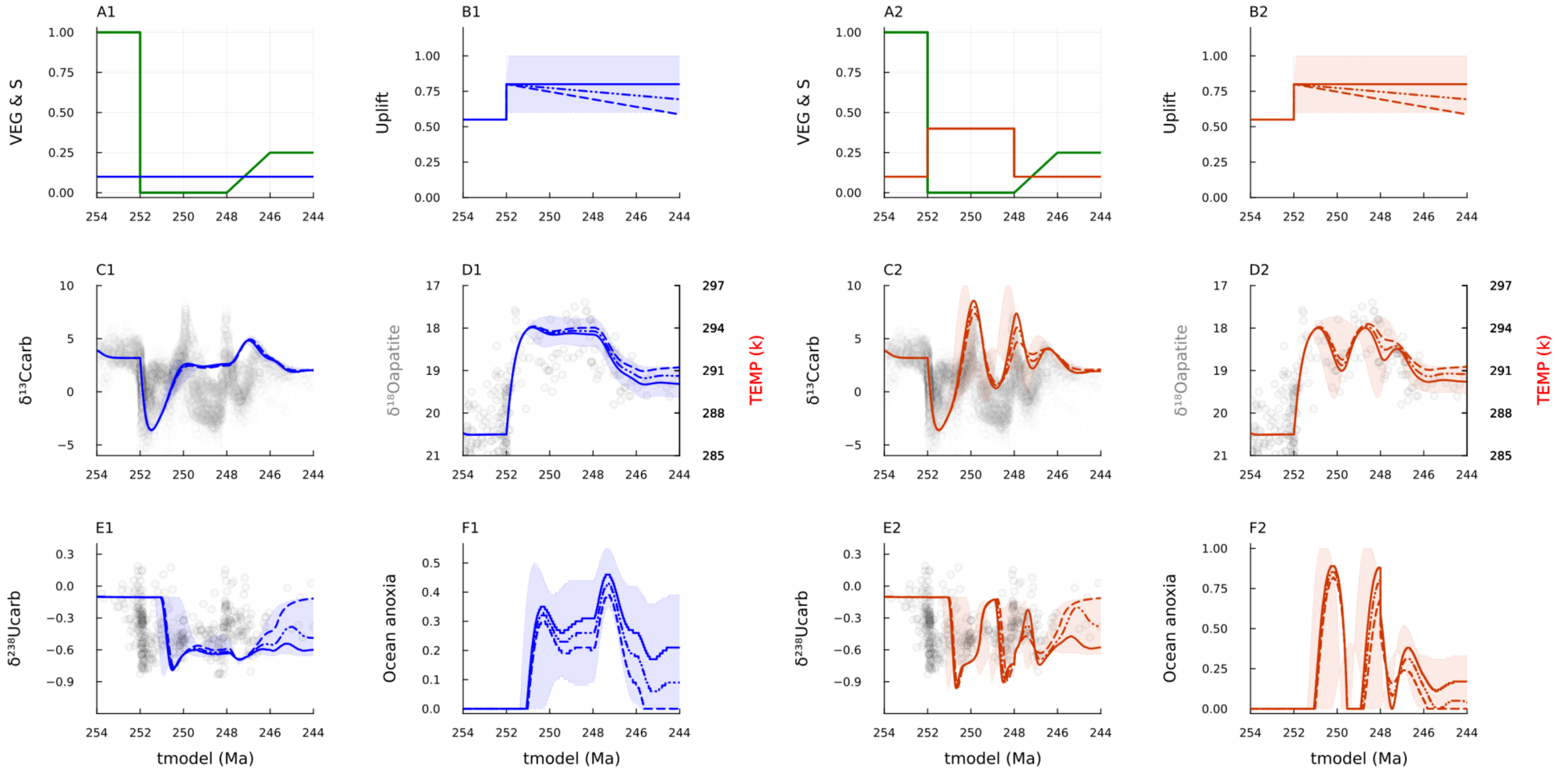

Figure S12. Sensitivity tests of the forcing Uplift. (A1-2) Vegetation (V, green line) and Stability forcings (stable system in blue, unstable system in red). (B1-2) Uplift forcing (U). Solid lines (U = 0.8) with color bars (U = [0.6, 1.0]), dash and dashdot lines represent Uplift values decay from early most Triassic (Uplift = 0.8) to 0.6 and 0.4 at 237Ma, respectively. (C-E) Model-data comparisons of $\delta^{13}C_{carb}$, $\delta^{18}O_{apatite}$ vs. Temperature (K), and $\delta^{238}U_{carb}$. (F1-2) Degree of ocean anoxia.

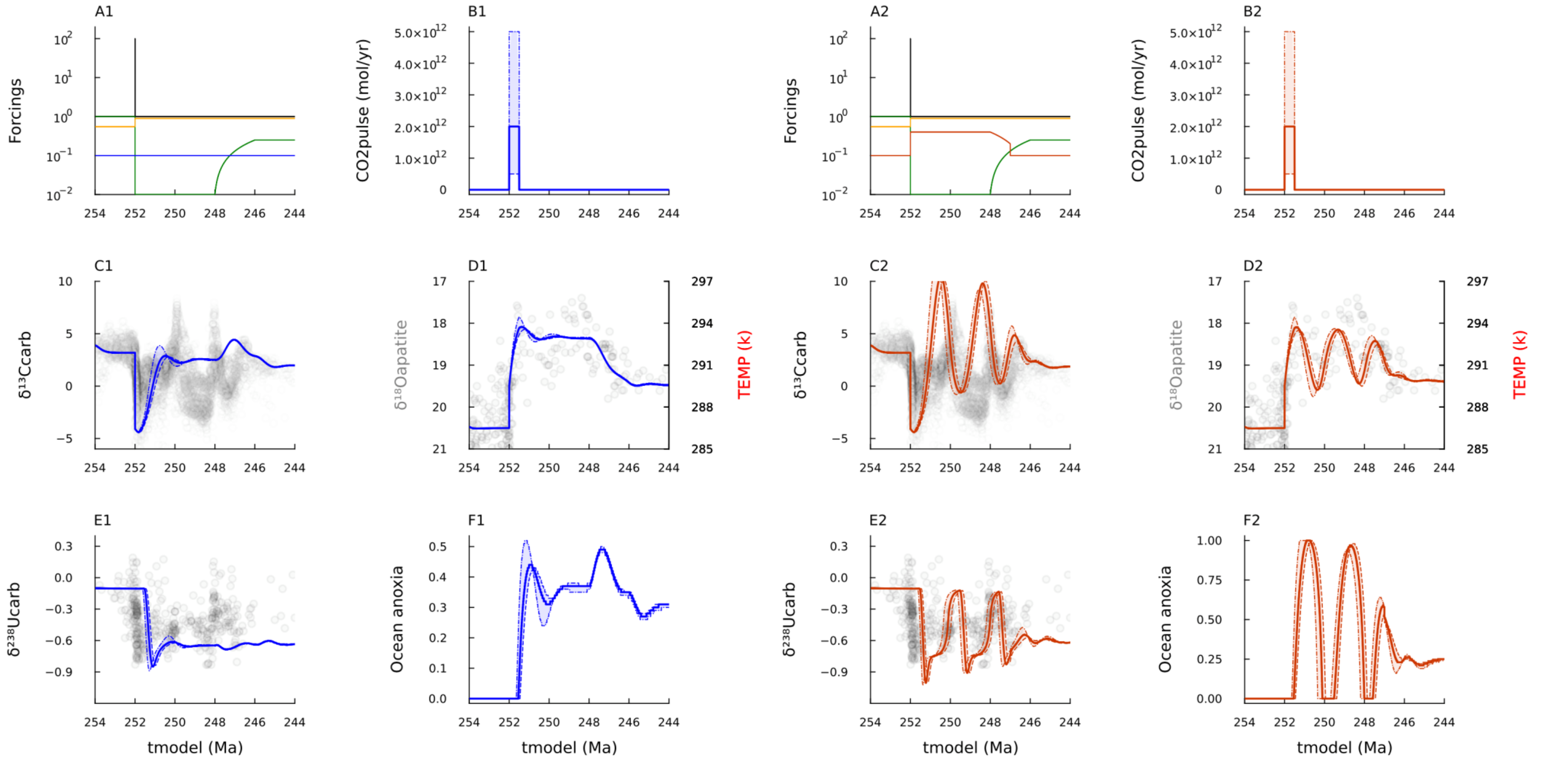

Figure S13. Extended model runs with input short-timescale pulses of oxidative weathering and CO_2. (A1-2) Forcings: VEG (green), Uplift (orange), S (blue for stable, red for unstable) and pulse of oxidative weathering (black). (B1-2) CO_2 pulses 0.5, 2.0, 5.0e12 mol/yr during 252 to 251.5 Ma. (C-E) Model-data comparisons of $\delta^{13}C_{carb}$, $\delta^{18}O_{apatite}$ vs. Temperature (K), and $\delta^{238}U_{carb}$. (F1-2) Degree of ocean anoxia.

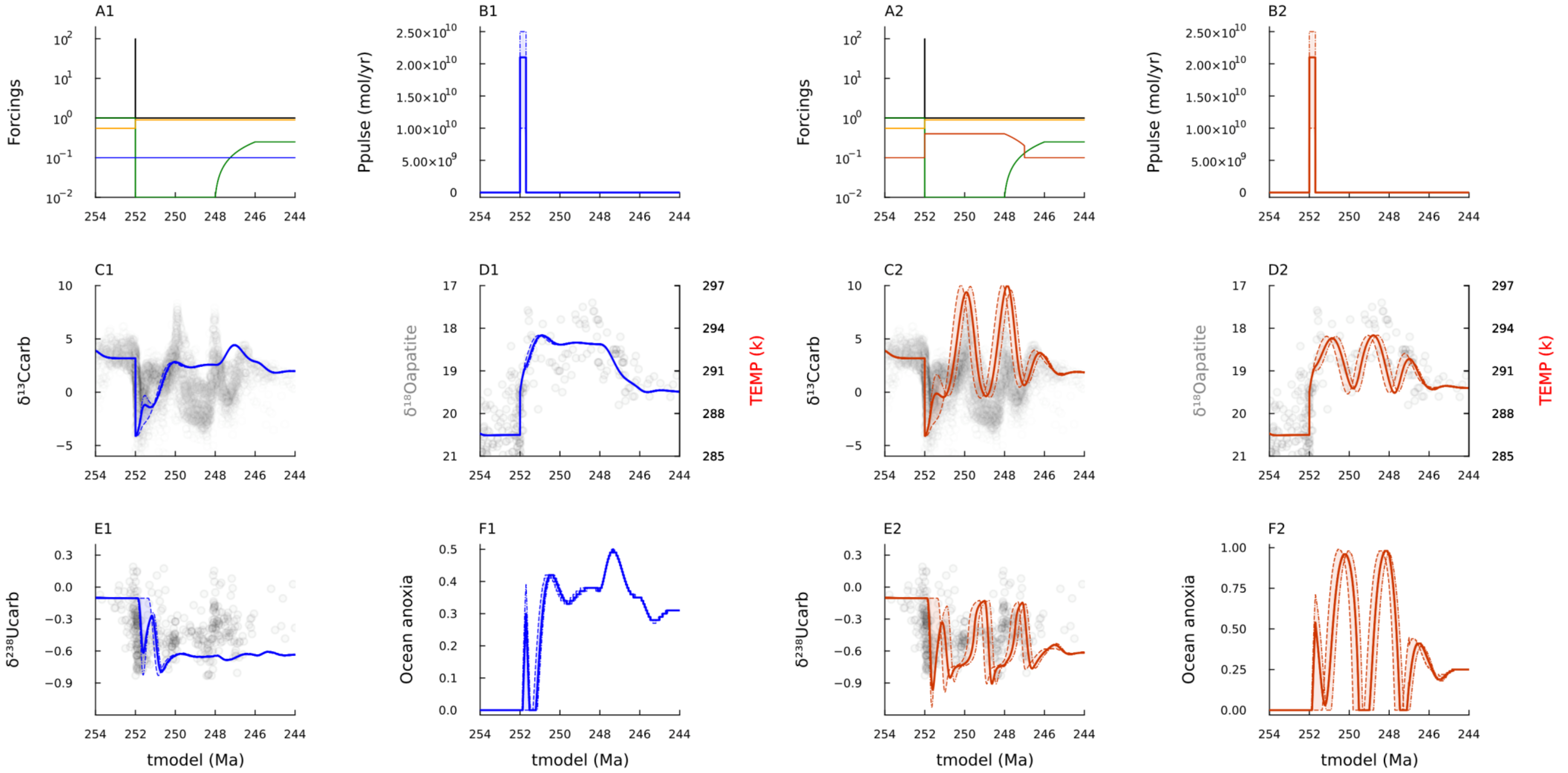

Figure S14. Extended model runs with short-timescale pulses of oxidative weathering and phosphorus. (A1-2) Forcings: VEG (green), Uplift (orange), S (blue for stable, red for unstable) and pulse of oxidative weathering (black). (B1-2) Phosphorus pulses 1.0, 2.1, 2.5e10 mol/yr during 252 to 251.7 Ma. (C-E) Model-data comparisons of $\delta^{13}C_{carb}$, $\delta^{18}O_{apatite}$ vs. Temperature (K), and $\delta^{238}U_{carb}$. (F1-2) Degree of ocean anoxia.

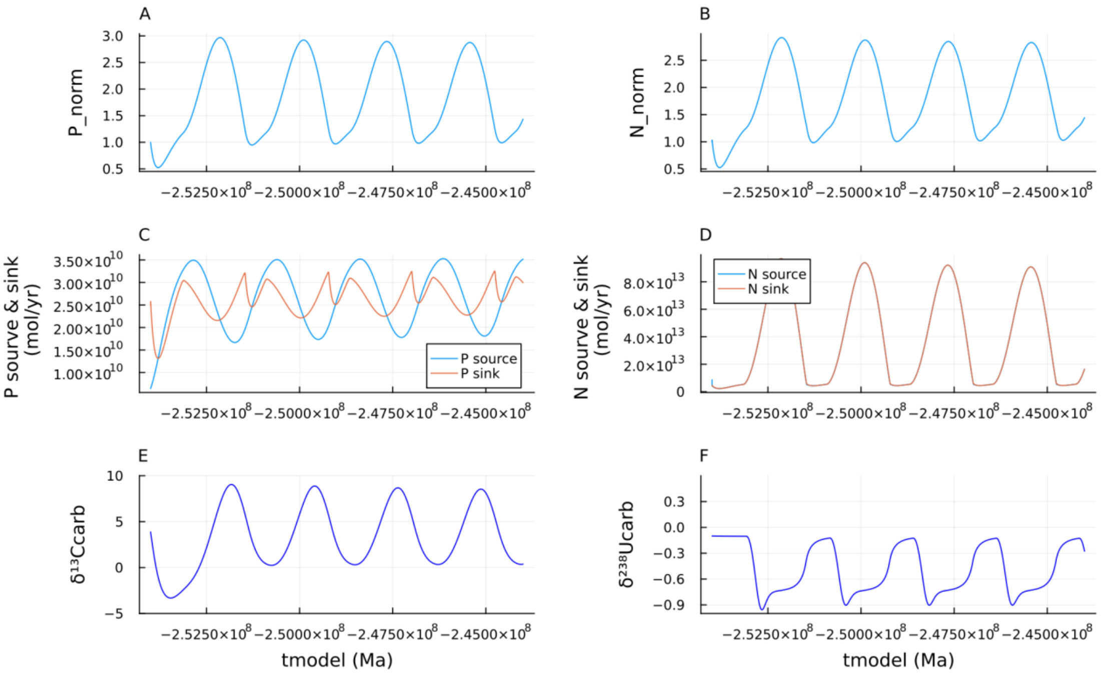

Figure S15. Extended model run shows source-sink dynamics of the nitrogen involved limit cycle system. (A-B) Normalized oceanic phosphorus and nitrogen levels. (C-D) Source (blue) and sink (orange) of the oceanic phosphorus and nitrogen. (E-F) Model predicted carbon and uranium isotope curves. Note that the forcings are set to constant: VEG = 0, S = 0.4, Uplift = 0.8.

Table S1. Data source of the reviewed $\delta^{13}C_{carb}$ and $\delta^{238}U_{carb}$ from 47 sections. We only include $\delta^{18}O_{apatite}$ data from South China to avoid the temperature offset between longitude or altitude[39-40].

No.	Sections	Locations		References
Carbon isotopes				
1	Shangsi	Sichuan, S.China	E.Paleotethys	Shen-2013, Riccardi-2007: http://dx.doi.org/10.1016/j.epsl.2013.05.020, do
2	Chaohu	Anhui, S.China	E.Paleotethys	Tong-2007: DOI: 10.1002/gj.1084
3	Hushan	Jiangsu, S.China	E.Paleotethys	Tong-2007: DOI: 10.1002/gj.1084
4	Jiarong	Guizhou, S.China	E.Paleotethys	Zhao-2019:https://doi.org/10.1016/j.palaeo.2019.109393
5	Meishan	Zhejiang, S.China	E.Paleotethys	Tong-2007, Burgess-2014: DOI: 10.1002/gj.1084, www.pnas.org/lookup/su
6	Guandao	Guizhou, S.China	E.Paleotethys	Meyer-2011, Lehrmann-2015:Meyer-2011:doi:10.1016/j.epsl.2010.12.03
7	Bianyang	Guizhou, S.China	E.Paleotethys	Meyer-2011:doi:10.1016/j.epsl.2010.12.033
8	Mingtang	Guizhou, S.China	E.Paleotethys	Song-2013:http://dx.doi.org/10.1016/j.gloplacha.2012.10.023
9	Shitouzhai	Guizhou, S.China	E.Paleotethys	Lyu-2019: https://doi.org/10.1016/j.earscirev.2019.01.010
10	Dukou	Sichuan, S.China	E.Paleotethys	Shen-2013: http://dx.doi.org/10.1016/j.epsl.2013.05.020
11	Zuodeng	Guangxi, S.China	E.Paleotethys	Tong-2007, Zhang-2019: DOI: 10.1002/gj.1084, https://doi.org/10.1016/j.e
12	Dawen	Guizhou, S.China	E.Paleotethys	Meyer-2011:doi:10.1016/j.epsl.2010.12.033
13	Dajiang	Guizhou, S.China	E.Paleotethys	Meyer-2011:doi:10.1016/j.epsl.2010.12.033
14	Yangou	Jiangxi, S.China	E.Paleotethys	Zhao-2021: https://doi.org/10.1016/j.gloplacha.2021.103583
15	Spiti Valley (Mud+Lingti+Guling)	Himachal Pradesh, India	S. Neotethys	Stebbins:https://doi.org/10.1016/j.earscirev.2018.09.007
16	Mud/Muth (spiti)	Himachal Pradesh, India	S. Neotethys	Sun-2021:https://doi.org/10.1016/j.gloplacha.2020.103363
17	Losar	Himachal Pradesh, India	S. Neotethys	Galfetti-2007a:doi:10.1016/j.palaeo.2006.08.014
18	Guryul Ravine	Kashmir, N. India	S. Neotethys	Wang-2019:https://doi.org/10.1016/j.epsl.2019.02.026
19	Salt & Surghar Range	Daud Khel, N. Pakistan	S. Neotethys	Hermann-2011:doi:10.1016/j.sedgeo.2010.11.003
20	Tulong	Tibet, China	S. Neotethys	Brühwiler-2009; An-2020:doi:10.1016/j.sedgeo.2009.10.003; doi.org/10.37
21	Kuh-e-Ali Bashi	Azerbaijan, NW.Iran	C.Paleotethys	Schobben-2014:http://dx.doi.org/10.1016/j.gr.2013.07.019
22	Zal	Azerbaijan, NW.Iran	C.Paleotethys	Zhang-2018: DOI: 10.1126/sciadv.1602921
23	Abadeh	Isfahan, C. Iran	C.Paleotethys	Horacek-2007a, Shen-2013, Chen-2020: doi:10.1016/j.palaeo.2006.11.05
24	Shahreza	Isfahan, C. Iran	C.Paleotethys	Korte-2004b
25	Kisejin	Abegarm, NW.Iran	C.Paleotethys	Maaleki-Moghadam-2019:https://doi.org/10.1016/j.palaeo.2018.12.007
26	Taškent	Aladag Nappe, Turkey	W. Tethys	Lau-2016: doi:10.1073/pnas.1515080113
27	Çürük dağ	Turkey	W. Tethys	Richoz-2006, Clarkson-2013: http://dx.doi.org/10.1016/j.gr.2012.10.002
28	Bálvány N & E	Bűkk, N. Hungary	W.Paleotethys	Schobben-2017:http://dx.doi.org/10.1016/j.palaeo.2017.02.025
29	Pufels/Bulla	S. Alps, Italy	W.Paleotethys	Horacek-2007b, Korte-2010: doi:10.1016/j.palaeo.2006.11.049, doi:10.101
30	L'Om Picol/Uomo	S. Alps, Italy	W.Paleotethys	Horacek-2007b: doi:10.1016/j.jseaes.2009.08.012
31	Lungenfrischgraben	S. Alps, Italy	W.Paleotethys	Horacek-2010:doi:10.1016/j.palaeo.2010.02.016
32	Trudener Bach	S. Alps, Italy	W.Paleotethys	Horacek-2010:doi:10.1016/j.palaeo.2010.02.016
33	GK-1	Southern Alps, Austria	W.Paleotethys	Zhang-2020:https://doi.org/10.1016/j.gca.2020.01.032
34	Plavno	S. Alps, Croatia	W.Paleotethys	Aljinović-2018:https://doi.org/10.1007/s12583-018-0787-3
35	Idrija–Žiri	Žiri, Slovenia	W.Paleotethys	Chen-2016: http://dx.doi.org/10.1016/j.palaeo.2015.12.013
36	Radio Tower	Oman	SW. Neotethys	Chen-2019:https://doi.org/10.1016/j.earscirev.2019.03.004
37	Wadi Maqam	Oman	SW. Neotethys	Richoz-2006, Clarkson-2013: http://dx.doi.org/10.1016/j.gr.2012.10.002
38	Wadi Wasit South	Oman	SW. Neotethys	Richoz-2006, Clarkson-2013: http://dx.doi.org/10.1016/j.gr.2012.10.002
39	Baid (Wadi Alwa)	Oman	SW. Neotethys	Richoz-2006, Clarkson-2013: http://dx.doi.org/10.1016/j.gr.2012.10.002
40	Wadi wasit	Oman	SW. Neotethys	Krystyn-2003:doi:10.1016/S0031-0182(02)00670-3
41	Wadi Sahtan	Oman	SW. Neotethys	Richoz-2006, Clarkson-2013: http://dx.doi.org/10.1016/j.gr.2012.10.002
42	Musandam	UAE	SW. Neotethys	Clarkson-2013: http://dx.doi.org/10.1016/j.gr.2012.10.002
43	Hot springs	Idaho, USA	W. Pangea	Caravaca-2017: http://dx.doi.org/10.1016/j.gloplacha.2017.05.005
44	Spruce mountain & Confusion range	W. USA	W. Pangea	Saltzman-2013:doi:10 1130/G33906 1
45	Mineral Mountains	W. USA	W. Pangea	Thomazo-2016:DOI: 10.1111/gbi.12174
46	Jesmond	British Columbia, Canada	mid-Panthalassa	Zhang-2019:https://doi.org/10.1016/j.earscirev.2018.10.012
47	Kamura	Shioinouso, Japan	Panthalassa	Zhang-2019, Horacek-2009:doi:10.1016/j.jseaes.2008.05.005, http://dx.dc
Uranium isotopes				
1	Jiarong	Guizhou, S.China	E.Paleotethys	Zhao-2019:https://doi.org/10.1016/j.palaeo.2019.109393
2	Zuodeng	Guangxi, S.China	E.Paleotethys	Zhang-2019: https://doi.org/10.1016/j.earscirev.2018.10.012
3	Dawen	Guizhou, S.China	E.Paleotethys	Brennecka-2011:doi/10.1073/pnas.1106039108
4	Guandao	Guizhou, S.China	E.Paleotethys	Lau-2016:doi/10.1073/pnas.1515080113
5	Dajiang	Guizhou, S.China	E.Paleotethys	Lau-2016:doi/10.1073/pnas.1515080113
6	Taškent	Aladag Nappe, Turkey	W. Tethys	Lau-2016: doi:10.1073/pnas.1515080113
7	Zal	Azerbaijan, NW.Iran	C.Paleotethys	Zhang-2018: DOI: 10.1126/sciadv.1602921
8	GK-1	Southern Alps, Austria	W.Paleotethys	Zhang-2020:https://doi.org/10.1016/j.gca.2020.01.032
9	Jesmond	British Columbia, Canada	mid-Panthalassa	Zhang-2019:https://doi.org/10.1016/j.earscirev.2018.10.012
10	Kamura	Shioinouso, Japan	Panthalassa	Zhang-2018:https://doi.org/10.1130/G39695.1

Table S2 End-Permian to Early Triassic conodonts associated age tied point[4].

Age tied points 年龄锚点 (Ma)	Bio-zones 生物地层	Boundary of stages (substages) 界限
237	Conod. Zone: *Paragondolella intermedia*	Ladinian-Carnian
241.46	Ammo. Zone: *Eoprotrachyceras curionii*	Anisian-Ladinian
244.99	Ammo. Zone: *Kocaella*	Aegean-Bithynian
246.8	Conod. Zone: *Chiosella timorensis*	Olenekian-Anisian
248.1	C. Zone: *Novispathodus. pingdingshanensis*	Smithian-Spathian
249.88	Conod. Zone: *Novispathodus waageni*	Induan-Olenekian
250.5	Conod. Zone: *Neospathodus Dieneri*	
250.9	Conod. Zone: *Sweetospathodus kummeli*	Griesbachian-Dieneran
251.87	Conod. Zone: *Isarcicella isarcica*	
251.902	Conod. Zone: *Hindeodus parvus*	Permian-Triassic
251.941	Mass extinction level (base of "Boundary Clay")	
251.951	Conod. Zone: *Clarkina meishanensis*	
252.064	Conod. Zone: *Clarkina yini*	
253.253	Conod. Zone: *Clarkina changxingensis*	
254.14	Conod. Zone: *Clarkina wangi*	Wuchiapingian-Changhsingian

Table S3 Atmosphere, climate and land model equations of the biogeochemical processes of the end-Permian stable system. GEOCARB*[64], COPSE*[45], COPSE**[46], and CPU[65].

Variable/Process	Equation	Units	References/Notes
Differential equations			
Ocean-atmosphere C	dA/dt = oxidw + carbw +ocdeg + ccdeg - mocb - locb - mccb	mol C/yr	GEOCARB*, COPSE**
Ocean-atmosphere O	dO/dt = mocb + locb - oxidw - ocdeg	mol C/yr	GEOCARB*, COPSE**
Ocean P balance	dP/dt = psea - mopb	mol C/yr	COPSE*,**
Ocean U balance	$dU/dt = F_{riv} - F_{anoxic} - F_{other}$		
C isotope balance	$d\delta A/dt = ((oxidw + ocdeg) * \delta_G + (carbw + ccdeg) * \delta_C - locb * \delta locb - mocb * \delta_{mocb} - mccb * \delta_{DIC})/A$	mol C/yr	COPSE*,**
U isotope balance	$d\delta U/dt = (F_{riv} * (\delta_{riv} - \delta_U) - F_{anoxic} * \Delta_{anoxic} - F_{other} * \Delta_{other})/U$	mol C/yr	CPU
Key variables			
Atmosphere pCO2	$pCO2 = (A/A_0)^2$	PAL	COPSE*,**
Global temperature relative to 15 °C	$\Delta T = kCO_2 * \ln(pCO_2) - k_{SL} * (age/600Ma)$	K	GEOCARB*, COPSE**
Pre-plant weathering pCO2 dependence	$f_{preplant} = k_{15} * (pCO_2)^{0.5}$	-	GEOCARB*, COPSE**
Kinetics granite temperature dependence	$f_{Tgran} = \exp(k_{Tgran} * \Delta T)$	-	COPSE**
Kinetics basalt temperature dependence	$f_{Tbas} = \exp(k_{Tbas} * \Delta T)$	-	COPSE**
Runoff silicate temperature dependence	$f_{runoff} = (1 + 0.038 * \Delta T)^{0.65}$	-	GEOCARB*, COPSE**
Runoff carbonate temperature dependence	$g_{runoff} = 1 + 0.087 * \Delta T$	-	GEOCARB*, COPSE**
Carbon fluxes			
Granite weathering	$granw = k_{granw} * U * a_{gran} * f_{Tgran} * f_{runoff} * f_{preplant}$	mol C/yr	GEOCARB*, COPSE**
Basalt weathering	$basw = k_{basw} * a_{bas} * f_{Tbas} * f_{runoff} * f_{preplant}$	mol C/yr	GEOCARB*, COPSE**
Silicate weathering	silw = granw + basw	mol C/yr	COPSE*,**
Carbonate weathering	$carbw = k_{14} * U * g_{runoff} * f_{preplant}$	mol C/yr	GEOCARB*, COPSE**
Organic C oxidation	$oxidw = k_{17} * U * (O/O_0)^{0.5}$	mol C/yr	COPSE*,**
Organic C degassing	$ocdeg = k_{13} * D$	mol C/yr	
Carbonate degassing	$ccdeg = k_{12} * D$	mol C/yr	
Terrestrial C_{org} burial	$locb = k_5 * CP_{land} * pland$	mol C/yr	
Phosphorus fluxes			
Fraction of P weathering from silicate	$P_{silw} = k_{Psilw} * silw/(k_{granw} + k_{basw})$	mol P/yr	COPSE*,**
Fraction of P weathering from carbonate	$P_{carbw} = k_{Pcarbw} * carbw/k_{14}$	mol P/yr	COPSE*,**
Fraction of P weathering from organic matter	$P_{oxidw} = k_{Poxidw} * oxidw/k_{17}$	mol P/yr	COPSE*,**
Reactive P weathering	$phosw = k_{10} * (P_{silw} + P_{carbw} + P_{oxidw})$	mol P/yr	COPSE*,**
Burial flux of P with land plant matter	$pland = k_{11} * V * phosw$	mol P/yr	COPSE*,**
Reactive P weathering to ocean	psea = phosw − pland	mol P/yr	COPSE*,**
Uranium fluxes			
U weathering (riverine input)	$F_{riv} = k_{riv} * silw/(k_{granw} + k_{basw})$	mol U/yr	CPU
Anoxic U sink	$F_{anoxic} = k_{anoxic} * (U/U_0) * f_{anoxic}/f_{anoxic0}$	mol U/yr	CPU, per-column
Other U sink	$F_{other} = k_{other} * (U/U_0) * (1 - f_{anoxic})/1 - f_{anoxic0})$	mol U/yr	CPU, per-column

Table S4 Physical forcings. Atmosphere, climate and land model parameters of the biogeochemical processes and constants of the end-Permian stable system.

Process/Forcing	Label	Value	Units	References/Notes
Model Forcing (at ~254 Ma)				
Metamorphic and volcanic degassing	D	1.13	-	COPSE**
Tectonic uplift	U	0.55	-	COPSE**
Apportioning of carbonate burial betwe	B	0.75	-	COPSE**
Normalised granite area	a_{gran}	1.56	-	COPSE**
Normalised basalt area	a_{bas}	0.7	-	COPSE**
Model climate				
Climate sensitivity control	k_{CO2}	4.328	˚C	GEOCARB*, COPSE**
Luminosity sensitivity control	k_{SL}	7.4	˚C	GEOCARB*, COPSE**
Carbon cycle				
Total atmosphere-ocean carbon preser	A_0	3.19E+18	mol	COPSE*,**
Carbonate C degassing	k_{12}	5.00E+12	mol/yr	to get dA/dt = 0
Organic C degassing	k_{13}	1.25E+12	mol/yr	COPSE*,**
Carbon isotope of sedimentary carbona	d13C_C	1.00	‰	to get d13C_DIC = 4‰
Carbon isotope of sedimentary organic	d13C_G	-22.0	‰	to get d13C_DIC = 4‰
Carbonate weathering	k_{14}	1.34E+13	mol/yr	COPSE*
Pre-plant weathering	k_{15}	0.15		COPSE*,**
Oxidative C weathering	k_{17}	7.75E+12	mol/yr	COPSE*
Temperature sensitivity of granite weatl	k_{Tgran}	0.09		COPSE*
Temperature sensitivity of basalt weath	k_{Tbas}	0.09		COPSE*
Granite weathering	k_{granw}	4.50E+12	mol/yr	COPSE**
Basalt weathering	k_{basw}	1.50E+12	mol/yr	COPSE**
Land organic Carbon burial	k_5	4.50E+12	mol/yr	COPSE**
Oxygen cycle				
Total atmosphere-ocean oxygen presei	O_0	3.70E+19	mol	COPSE*,**
Phosphorus cycle				
Total ocean phosphorus present-day va	P_0	3.10E+15	mol	COPSE*,**
Reactive P weathering	k_{10}	3.90E+10	mol/yr	COPSE*,**
Silicates fraction of P weathering	k_{Psilw}	1.0		COPSE**
Carbonates fraction of P weathering	k_{Pcarbw}	0.00		COPSE**
Oxidative fraction of P weathering	k_{Poxidw}	0.00		COPSE**
Land C to P ratio	CP_{land}	1000.00		to get dO/dt = 0
Fraction of weathering P burial on land	k_{11}	0.10		to get dO/dt = 0
Uranium cycle				
Total ocean uranium present-day value	U_0	1.90E+13	mol	CPU
U weathering (along sliw)	k_{Usilw}	3.00E+07	mol/yr	CPU

Note: Unless stated above, all the other COPSE forcings are constant 1.0

9. Supplementary references

1. Veizer, J., Holser, W.T., Wilgus, C.K., 1980. Correlation of $^{13}C/^{12}C$ and $^{34}S/^{32}S$ secular variations. Geochimica et Cosmochimica Acta 44, 579–587.
2. Kaufman, A. J., Hayes, J. M., Knoll, A. H. and Germs, G. J. B., 1991. Isotopic compositions of carbonates and organic carbon from upper Proterozoic successions in Namibia: stratigraphic variation and the effects of diagenesis and metamorphism. Precambrian Research 49, 301–327.
3. Cramer, B.D. and Jarvis, I. 2020. Chapter 11 - Carbon Isotope Stratigraphy. In: Gradstein, F.M., Ogg, J.G., Schmitz, M.D., Ogg, G.M. (Eds.) The Geologic Time Scale 2020. Elsevier, Amsterdam, 309–343.
4. Ogg, J. G. and Chen, Z. Q., 2020. Chapter 25. The Triassic period. Geologic Time Scale 2020. Elsevier Publisher, ISBN: 978-0128243602.
5. Li, Z. H., Guo, Z., Chen, Z. Q. et al., 2021. A novel carbon cycle turbulence index identifies environmental and ecological perturbations. Geochemical Perspectives Letters 20, 11-15.
6. Lyu, Z., Zhang, L., Algeo, T. J., Zhao, L., Chen, Z. Q., Li, C., Ma, B. and Ye, F., 2019. Global-ocean circulation changes during the Smithian–Spathian transition inferred from carbon-sulfur cycle records. Earth-Science Reviews 195, 114–132.
7. Shen, S. Z, Cao, C. Q, et al., 2013. High-resolution $\delta^{13}C_{carb}$ chemostratigraphy from latest Guadalupian through earliest Triassic in South China and Iran. Earth and Planetary Science Letters 375, 156–165.
8. Tong, J. N., Zuo, J. X. and Chen, Z.,Q., 2007. Early Triassic carbon isotope excursions from South China: Proxies for devastation and restoration of marine ecosystems following the end-Permian mass extinction. Geological Journal 42, 371–389.
9. Zhao, H., Lyu, Z., Chen, Z. Q., Algeo, T. J., Orchard, M. J., Liu, Y., Hu, Z., Zhang, L. and Zhang, X., 2021. Integrated biochemostratigraphy of the Permian-Triassic boundary beds in a shallow carbonate platform setting (Yangou, South China). Global and Planetary Change 206, 103583.
10. Meyer, K. M., Yu, M., Jost, A. B., Kelley, B. M. and Payne, J. L. 2011. $\delta^{13}C$ evidence that high primary productivity delayed recovery from end-Permian mass extinction. Earth and Planetary Science Letters 302, 378–384.

11. Song, H., Wignall, P. B., Tong, J. and Yin, H. 2013. Two pulses of extinction during the Permian-Triassic crisis. Nature Geoscience 6, 52–56.
12. Brühwiler, T., Goudemand, N., Galfetti, T., Bucher, H., Baud, A., Ware, D., Hermann, E., Hochuli, P. A. and Martini, R. 2009. The Lower Triassic sedimentary and carbon isotope records from Tulong (South Tibet) and their significance for Tethyan palaeoceanography. Sedimentary Geology 222, 314–332.
13. Galfetti, T., Bucher, H., Brayard, A., Hochuli, P. A., Weissert, H., Guodun, K., Atudorei, V. and Guex, J., 2007. Late Early Triassic climate change: Insights from carbonate carbon isotopes, sedimentary evolution and ammonoid paleobiogeography. Palaeogeography, Palaeoclimatology, Palaeoecology 243, 394–411.
14. Stebbins, A., Algeo, T. J., Olsen, C., Sano, H., Rowe, H. and Hannigan, R., 2019. Sulfur-isotope evidence for recovery of seawater sulfate concentrations from a PTB minimum by the Smithian-Spathian transition. Earth-Science Reviews 195, 83–95.
15. Sun, Y. D., Richoz, S., Krystyn, L., Grasby, S. E., Chen, Y. L., Banerjee, D. and Joachimski, M. M. 2021. Integrated bio-chemostratigraphy of Lower and Middle Triassic marine successions at Spiti in the Indian Himalaya: Implications for the Early Triassic nutrient crisis. Global and Planetary Change 196, 103363.
16. Wang, X., Cawood, P. A., Zhao, H., Zhao, L., Grasby, S. E., Chen, Z. Q. and Zhang, L. 2019. Global mercury cycle during the end-Permian mass extinction and subsequent Early Triassic recovery. Earth and Planetary Science Letters 513, 144–155.
17. Hermann, E., Hochuli, P. A., Méhay S., Bucher, H., Brühwiler, T., Ware, D., Hautmann M., Roohi, G., ur-Rehman, K. and Yaseen, A., 2011. Organic matter and palaeoenvironmental signals during the Early Triassic biotic recovery: The Salt Range and Surghar Range records. Sedimentary Geology 234, 19–41.
18. Schobben, M., Ullmann, C. V., Leda, L., Korn, D., Struck, U., Reimold, W. U., Ghaderi, A., Algeo, T. J. and Korte, C., 2016. Discerning primary versus diagenetic signals in carbonate carbon and oxygen isotope records: An example from the Permian-Triassic boundary of Iran. Chemical Geology 422, 94–107.
19. Zhang, F., Romaniello, S.J., Algeo, T.J., Lau, K.V., Clapham, M.E., Richoz, S., Herrmann, A.D., Smith, H., Horacek, M. and Anbar, A.D., 2018a. Multiple

episodes of extensive marine anoxia linked to global warming and continental weathering following the latest Permian mass extinction. Science Advances 4(4), e1602921.

20. Horacek, M., Richoz, S., Brandner, R., Krystyn, L. and Spötl, C. 2007b. Evidence for recurrent changes in Lower Triassic oceanic circulation of the Tethys: The $\delta^{13}C$ record from marine sections in Iran. Palaeogeography, Palaeoclimatology, Palaeoecology 252, 355–369.
21. Korte, C., Kozur, H. W., Joachimski, M. M., Strauss, H., Veizer, J. and Schwark, L., 2004. Carbon, sulfur, oxygen and strontium isotope records, organic geochemistry and biostratigraphy across the Permian/Triassic boundary in Abadeh, Iran. International Journal of Earth Sciences 93, 565–581.
22. Maaleki-Moghadam, M., Rafiei, B., Richoz, S., Woods, A. D. and Krystyn, L., 2019. Anachronistic facies and carbon isotopes during the end-Permian biocrisis: Evidence from the mid-Tethys (Kisejin, Iran). Palaeogeography, Palaeoclimatology, Palaeoecology 516, 364–383.
23. Lau, K. V., Maher, K., Altiner, D., Kelley, B. M., Kump, L. R., Lehrmann, D. J., Silva-Tamayo, J. C., Weaver, K. L., Yu, M. and Payne, J. L., 2016. Marine anoxia and delayed Earth system recovery after the end-Permian extinction. Proceedings of the National Academy of Sciences of the United States of America 113, 2360–2365.
24. Schobben, M., Stebbins, A., Algeo, T. J., Strauss, H., Leda, L., Haas, J., Struck, U., Korn, D. and Korte, C., 2017. Volatile earliest Triassic sulfur cycle: A consequence of persistent low seawater sulfate concentrations and a high sulfur cycle turnover rate? Palaeogeography, Palaeoclimatology, Palaeoecology 486, 74–85.
25. Horacek, M., Brandner, R. and Abart, R., 2007a. Carbon isotope record of the P/T boundary and the Lower Triassic in the Southern Alps: Evidence for rapid changes in storage of organic carbon. Palaeogeography, Palaeoclimatology, Palaeoecology 252, 347–354.
26. Zhang, F., Shen, S., Cui, Y., Lenton, T. M., Dahl, T. W., Zhang, H., Zheng, Q., Wang, W., Krainer, K. and Anbar, A. D. 2020. Two distinct episodes of marine anoxia during the Permian-Triassic crisis evidenced by uranium isotopes in marine dolostones. Geochimica et Cosmochimica Acta 287, 165–179.

27. Aljinović, D., Horacek, M., Krystyn, L., Richoz, S., Kolar-Jurkovšek, T., Smirčić, D. and Jurkovšek, B., 2018. Western Tethyan epeiric ramp setting in the Early Triassic: an example from the Central Dinarides (Croatia). Journal of Earth Science 29, 806-823.
28. Chen, Y., Kolar-Jurkovšek, T., Jurkovšek, B., Aljinović, D. and Richoz, S., 2016. Early Triassic conodonts and carbonate carbon isotope record of the Idrija-Žiri area, Slovenia. Palaeogeography, Palaeoclimatology, Palaeoecology 444, 84–100.
29. Chen, Y., Richoz, S., Krystyn, L. and Zhang, Z., 2019. Quantitative stratigraphic correlation of Tethyan conodonts across the Smithian-Spathian (Early Triassic) extinction event. Earth-Science Reviews 195, 37–51.
30. Krystyn, L., Richoz, S., Baud, A. and Twitchett, R. J., 2003. A unique Permian-Triassic boundary section from the Neotethyan Hawasina Basin, Central Oman Mountains. Palaeogeography, Palaeoclimatology, Palaeoecology 191, 329–344.
31. Clarkson, M. O., Richoz, S., Wood, R. A., Maurer, F., Krystyn, L., McGurty, D. J. and Astratti, D. 2013. A new high-resolution δ13C record for the Early Triassic: Insights from the Arabian Platform. Gondwana Research 24, 233–242.
32. Zhang, F., Algeo, T. J., Romaniello, S. J., Cui Y., Zhao, L., Chen, Z. Q. and Anbar, A. D. 2018b. Congruent Permian-Triassic $\delta^{238}U$ records at Panthalassic and Tethyan sites: Confirmation of global-oceanic anoxia and validation of the U-isotope paleoredox proxy. Geology 46, 327–330.
33. Caravaca, G., Brayard, A., Vennin, E., Guiraud, M., Le Pourhiet, L., Grosjean, A. S., Thomazo, C., Olivier, N., Fara, E., Escarguel, G., Bylund, K. G., Jenks, J. F. and Stephen, D. A. 2018. Controlling factors for differential subsidence in the Sonoma Foreland Basin (Early Triassic, western USA). Geological Magazine 155, 1305–1329.
34. Saltzman, M. R. and Sedlacek, A. R. C., 2013. Chemostratigraphy indicates a relatively complete Late Permian to Early Triassic sequence in the western United States. Geology 41, 399–402.
35. Thomazo, C., Vennin, E., Brayard, A., Bour, I., Mathieu, O., Elmeknassi, S., Olivier, N., Escarguel, G., Bylund, K. G., Jenks, J., Stephen, D. A. and Fara, E., 2016. A diagenetic control on the Early Triassic Smithian-Spathian carbon

isotopic excursions recorded in the marine settings of the Thaynes Group (Utah, USA). Geobiology 14, 220–236.

36. Zhang, F., Algeo, T. J., Cui, Y., Shen, J., Song, H., Sano, H., Rowe, H. D. and Anbar, A. D. 2019. Global-ocean redox variations across the Smithian-Spathian boundary linked to concurrent climatic and biotic changes. Earth-Science Reviews 195, 147–168.
37. Brennecka, G. A., Herrmann, A. D., Algeo, T. J. and Anbar, A. D. 2011. Rapid expansion of oceanic anoxia immediately before the end-Permian mass extinction. Proceedings of the National Academy of Sciences 108, 17631–17634.
38. Zhao, H., Algeo, T. J., Liu, Y., Chen, Z. Q., Zhang, L., Hu, Z. and Li, Z., 2020. Lower Triassic carbonate δ238U record demonstrates expanded oceanic anoxia during Smithian Thermal Maximum and improved ventilation during Smithian-Spathian boundary cooling event. Palaeogeography, Palaeoclimatology, Palaeoecology, 539, 109393.
39. Joachimski, M. M., Lai, X., Shen, S., Jiang, H., Luo, G., Chen, B., Chen, J. and Sun, Y. D., 2012. Climate warming in the latest Permian and the Permian-Triassic mass extinction. Geology 40, 195–198.
40. Sun, Y. D., Joachimski, M. M., Wignall, P. B., Yan, C., Chen, Y., Jiang, H., Wang, L. and Lai, X. 2012. Lethally Hot Temperatures During the Early Triassic Greenhouse. Science 338, 366–370.
41. Trotter, J. A., Williams, I. S., Nicora, A., Mazza, M. and Rigo, M. 2015. Long-term cycles of Triassic climate change: A new δ18O record from conodont apatite. Earth and Planetary Science Letters 415, 165–174.
42. Song, H., Wignall, P. B., Song, H., Dai, X. and Chu, D., 2019. Seawater temperature and dissolved oxygen over the past 500 million years. Journal of Earth Science, 30, 236-243.
43. Daines, S. J. and Li, Z., 2024. Excitable Dynamics of Neoproterozoic to Early Paleozoic Atmospheric and Ocean Oxygen. Preprint at https://doi.org/10.31223/X52T4T.
44. Canfield, D. E., 1998. A new model for Proterozoic ocean chemistry. Nature 396, 450–453.
45. Bergman, N. M., Lenton, T. M. and Watson, A. J., 2004. COPSE: A new model of biogeochemical cycling over phanerozoic time. American Journal of Science 304, 397–437.

46. Lenton, T. M., Daines, S. J. and Mills, B. J. W., 2018. COPSE reloaded: An improved model of biogeochemical cycling over Phanerozoic time. Earth-Science Reviews 178, 1–28.
47. Van Cappellen, P. and Ingall, E. D. 1996. Redox Stabilization of the Atmosphere and Oceans by Phosphorus-Limited Marine Productivity. Science 271, 493–496.
48. Slomp, C. P. and Van Cappellen, P. 2007. The global marine phosphorus cycle: sensitivity to oceanic circulation. Biogeosciences, 4(2), 155-171.
49. Alcott, L. J., Mills, B. J. W. and Poulton, S. W., 2019. Stepwise Earth oxygenation is an inherent property of global biogeochemical cycling. Science 366, 1333–1337.
50. Algeo, T.J. and Ingall, E., 2007. Sedimentary Corg: P ratios, paleocean ventilation, and Phanerozoic atmospheric pO2. Palaeogeography, Palaeoclimatology, Palaeoecology, 256(3-4), pp.130-155.
51. Lenton, T.M., Watson, A.J., 2000a. Redfield revisited: 1. Regulation of nitrate, phosphate, and oxygen in the ocean. Glob. Biogeochem. Cycles 14, 225–248.
52. Lenton, T.M., Watson, A.J., 2000b. Redfield revisited: 2. What regulates the oxygen content of the atmosphere? Glob. Biogeochem. Cycles 14, 249–268.
53. Dal Corso, J., Mills, B. J. W., Chu, D., Newton, R. J. and Song, H., 2022. Background Earth system state amplified Carnian (Late Triassic) environmental changes. Earth and Planetary Science Letters 578, 117321.
54. Dal Corso, J., Mills, B. J., Chu, D., Newton, R. J., Mather, T. A., Shu, W., Wu, Y., Tong, J. and Wignall, P. B., 2020. Permo–Triassic boundary carbon and mercury cycling linked to terrestrial ecosystem collapse. Nature Communications 11, 2962.
55. Payne, J. L. and Kump, L. R., 2007. Evidence for recurrent Early Triassic massive volcanism from quantitative interpretation of carbon isotope fluctuations. Earth and Planetary Science Letters, 256(1-2), 264-277.
56. Shen, W., Sun, Y., Lin, Y., Liu, D. and Chai, P., 2011. Evidence for wildfire in the Meishan section and implications for Permian–Triassic events. Geochimica et Cosmochimica Acta, 75(7), 1992-2006.
57. Benton, M. J. and Newell, A. J., 2014. Impacts of global warming on Permo-Triassic terrestrial ecosystems. Gondwana Research 25, 1308-1337.

58. Grasby, S. E., Shen, W., Yin, R., Gleason, J. D., Blum, J. D., Lepak, R. F., Hurley, J. P. and Beauchamp, B., 2017. Isotopic signatures of mercury contamination in latest Permian oceans. Geology 45, 55-58.
59. Liu, D., Zhou, C., Keesing, J.K. et al., 2022. Wildfires enhance phytoplankton production in tropical oceans. Nature Communications 13, 1348.
60. Wang, Y., Chen, H. H., Tang, R., He, D., Lee, Z., Xue, H., Wells, M., Boss, E. and Chai, F., 2022. Australian fire nourishes ocean phytoplankton bloom. Science of the Total Environment 807, 150775.
61. Chen, Z.Q., Fang, Y., Wignall, P.B., Guo, Z., Wu, S., Liu, Z., Wang, R., Huang, Y. and Feng, X., 2022. Microbial blooms triggered pyrite framboid enrichment and oxygen depletion in carbonate platforms immediately after the latest Permian extinction. Geophysical Research Letters, 49(7), p.e2021GL096998.
62. Aksnes, D. L. and Egge, J. K., 1991. A theoretical model for nutrient uptake in phytoplankton. Marine ecology progress series. Oldendorf 70, 65-72.
63. Kriest, I. and Oschlies, A., 2007. Modelling the effect of cell-size-dependent nutrient uptake and exudation on phytoplankton size spectra. Deep Sea Research Part I: Oceanographic Research Papers, 54(9), pp.1593-1618.
64. Berner, R. A. 2006. GEOCARBSULF: A combined model for Phanerozoic atmospheric O2 and CO2. Geochimica et Cosmochimica Acta 70, 5653–5664.
65. Zhang, F., Lenton, T.M., del Rey, Á., Romaniello, S.J., Chen, X., Planavsky, N.J., Clarkson, M.O., Dahl, T.W., Lau, K.V., Wang, W. and Li, Z., 2020. Uranium isotopes in marine carbonates as a global ocean paleoredox proxy: a critical review. Geochimica et Cosmochimica Acta, 287, pp.27-49.
