# Supplementary material for: Earth system instability amplified biogeochemical oscillations following the end-Permian mass extinction
Source: Nat Commun. 2025 Apr 18;16:3703. doi: 10.1038/s41467-025-59038-0 (PMC12008425; doi:10.1038/s41467-025-59038-0)
Supplement: Supplementary file 2 — Description of Additional Supplementary Files [file 41467_2025_59038_MOESM2_ESM.pdf]

Description of Additional Supplementary Files:

Supplementary Data 1: The raw datum used to illustrate Figure 1. The first sheet: age tied points; The second sheet: the datum source of carbon and uranium isotopes, see the source of oxygen isotope in Table S1; The third sheet: per-section carbon isotopes; The 4th to 6th sheets: the carbon-uranium-oxygen isotope data sorted chronologically.
